# Supplementary figures and images for: Refining Genotypes and Phenotypes in KCNA2-Related Neurological Disorders
Source: Int J Mol Sci. 2021 Mar 10;22(6):2824. doi: 10.3390/ijms22062824 (PMC7999221; doi:10.3390/ijms22062824)

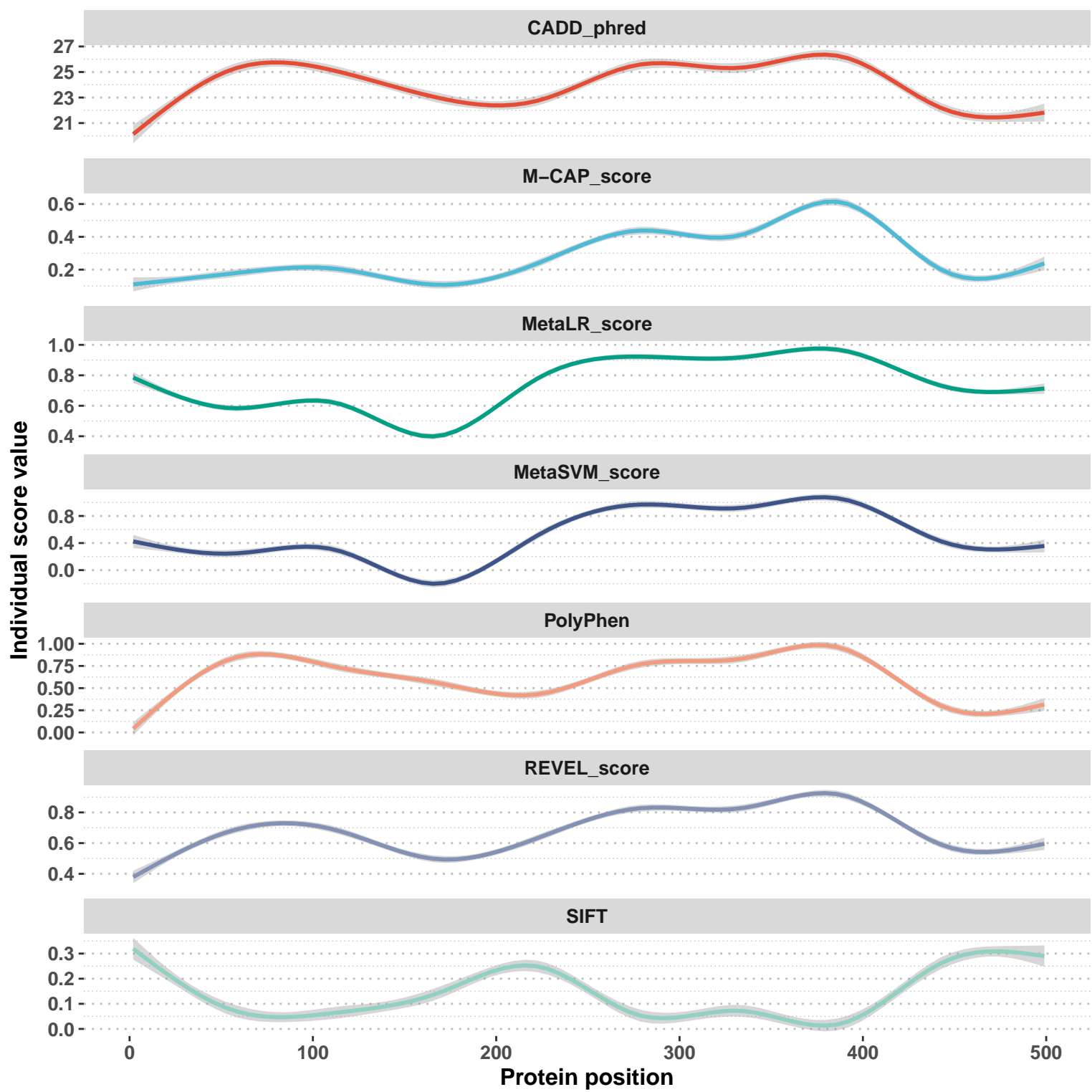

Supplement: Supplementary file 1 [file ijms-22-02824-s001.zip › Supplement/Supplement_Figure_S1_Synopsis of all annotated VEP scores.pdf]
